# Respiratory Rate Recovery After Submaximal Lunging Exercise Is Delayed in Asthmatic Horses with Neutrophilic Airway Inflammation

**DOI:** 10.3390/ani15050713

**Published:** 2025-03-02

**Authors:** Julia Röschmann, Jan Naef, Camille Doras, Vinzenz Gerber

**Affiliations:** 1 Swiss Institute of Equine Medicine (ISME), Department of Clinical Veterinary Medicine, Vetsuisse-Faculty, University of Bern, 3012 Bern, Switzerland; 2 Veterinary Public Health Institute (VPHI), Vetsuisse-Faculty, University of Bern, 3012 Bern, Switzerland

**Keywords:** equine asthma, bronchoalveolar lavage, exercise testing, decreased performance

## Abstract

Equine asthma is a common cause of poor performance, but little is known about its impact on submaximal exercise. The aim of the present study was to evaluate the effects of equine asthma and potential confounding factors on recovery of respiratory rate (RR) and heart rate (HR) after a lunging exercise test. Thirty-seven horses completed a standardized 15 min lunging exercise including HR and RR recording pre- and up to 30 min post-exercise and venous blood lactate measurement. Endoscopy was performed and bronchoalveolar lavage fluid cytology was used to categorize horses as controls or asthmatics. In the univariate analyses, asthmatic horses (*n* = 21) showed prolonged RR recovery (OR 52.25, 95% CI 5.17–528.28, *p* = 0.001) and took more than 15 min to return to pre-exercise levels. Prolonged recovery was associated with bronchoalveolar lavage fluid neutrophilia (OR 1.14, 95% CI 1.01–1.29, *p* = 0.04) and also with trot velocity (OR 17.32, 95% CI 1.23–244.15, *p* = 0.03). HR recovery increased with higher training level (OR 0.14, 95% CI 0.02–0.83, *p* = 0.03) and decreased with age (OR 1.20, 95% CI 1.03–1.40, *p* = 0.02). RR recovery after the submaximal lunging exercise could offer a practical test for functional deficits associated with equine asthma.

## Simple Summary

Equine asthma is a common respiratory condition that can affect horses’ athletic performance by impairing lung function. Severe cases may present with noticeable respiratory signs, but mild-to-moderate asthma can remain undetected at rest. Its impact on performance, particularly in non-racehorses used for leisure or sport, is still poorly understood. This study investigates how equine asthma influences the recovery of heart rate (HR) and respiratory rate (RR) following a standardized submaximal lunging exercise in 37 horses of different breeds and training levels. Recovery of RR, but not HR, was significantly delayed in horses with bronchoalveolar lavage fluid neutrophilia, a common type of airway inflammation in equine asthma. Tracheal mucus accumulation or elevated airway eosinophils and mast cells did not influence RR recovery. HR recovery time decreased with higher training level and increased with age, suggesting that cardiovascular fitness plays a larger role in HR response. These findings highlight the potential of submaximal lunging exercise testing as a practical tool for identifying respiratory limitations in asthmatic leisure and sport horses.

## 1. Introduction

Equine asthma can be a cause of poor performance even in mild-to-moderate cases that show no or few clinical signs at rest [1]. While practical submaximal testing could be more relevant in leisure and sport horses, the impact of equine asthma on performance parameters has mostly been investigated using high-intensity exercise tests. In standardbred and thoroughbred racehorses, several studies using intensive exercise protocols have demonstrated that mild degrees of equine asthma, defined by bronchoalveolar lavage fluid (BALF) cytology, can impair exercise capacity [2,3,4,5,6,7].

Increased mast cells and neutrophils in BALF were associated with reduced racing performance [8]. Incremental maximal workload treadmill tests showed impaired athletic capacity with a decreased speed at the lactate level of 4 mmol/l in standardbred racehorses [4]. Using a similar exercise test, one study showed that increased airway neutrophilia negatively affects performance [5]. A ridden exercise test at maximal speed revealed differences in lung function between healthy and asthmatic thoroughbred racehorses 15 min post-exercise using respiratory oscillometry [6]. Furthermore, Fraipont et al. reported an association of poor performance with higher neutrophil percentages in BALF in endurance horses after a treadmill test [9].

Only a few studies have investigated post-exercise parameters during the recovery phase. One study on respiratory mechanics in standardbred and thoroughbred racehorses presenting poor athletic performance demonstrated altered pulmonary function parameters immediately post-exercise associated with higher neutrophil percentages in tracheal wash samples after an intensive treadmill exercise [10]. A maximal ridden exercise test revealed lung function deficits 15 min post-exercise in asthmatic thoroughbred horses [6]. Conversely, there were no associations of cytological signs of asthma with heart rate (HR), respiratory rate (RR) and body temperature in asymptomatic pacing horses 30 min after performing an intense ridden exercise [11], suggesting that effects of subclinical asthma might be insignificant for recovery or may go undetected without targeted submaximal testing.

Exercise intolerance and delayed recovery are often reported by owners of sport and leisure horses but is difficult to assess objectively [12], since validated tests for the effects of equine asthma in non-racehorses are largely lacking. A submaximal lunging exercise test has been recommended to assess recovery of HR and RR at 5 min intervals for up to 30 min after completion of the exercise. Healthy horses are expected to reach pre-exercise values within 15 min. Despite being used in clinical practice, this simple test is merely described in a German textbook [13]. It has not been critically investigated for its potential to detect asthma-related delayed HR and RR recovery. Yet, one study using such a submaximal lunging exercise protocol, consisting of 5 min walking, 5 min of trot and 5 min of canter, reported post-exercise differences in lung function in asthmatic compared to control horses. Electrical impedance tomography-derived peak inspiratory and expiratory flows were increased in asthmatic horses 15 min after exercise, but not before exercise. However, neither HR nor RR were described during the exercise and recovery phase [14].

The present study aimed to determine whether equine asthma prolongs post-exercise HR and RR recovery following submaximal lunging exercise. Horses were characterized using a standardized clinical score, BALF cytology and mucus accumulation in the trachea. Potential confounding factors, such as signalment, excitement (agitation), training level, body condition, velocity at each gait phase, post-exercise venous lactate levels, as well as extrinsic factors (ambient temperature and humidity) were also assessed. We hypothesized that asthma status based on BALF cytology prolongs RR and HR recovery after standardized submaximal exercise. Specifically, this study investigated whether recovery is delayed in asthmatic horses (i.e., taking >15 min to return to pre-exercise values) while accounting for intrinsic and extrinsic confounding factors.

## 2. Materials and Methods

### 2.1. Study Design and Animals

This prospective, observational, analytical cross-sectional study included horses presented for evaluation of exercise tolerance and/or signs of lower respiratory tract disease. Horses were required to complete the full lunging exercise test including all three gait phases (walk, trot, canter) with a good fit of the monitoring device. Exclusion criteria included a history of recurrent laryngeal neuropathy, exercise-induced pulmonary hemorrhage or exertional myopathy; cardiac arrhythmias other than a second-degree AV-block on cardiac auscultation; lameness or abnormal upper airway noises during lunging; endoscopic signs of laryngeal asymmetry compatible with recurrent laryngeal neuropathy or presence of blood in the trachea post-exercise. Also, any medication had to be discontinued at least 24 h before the exercise test. Data were collected between March and October 2024. All procedures were performed by one of the authors (JR), who was assisted by attending clinicians for endoscopy and BALF collection. The experimental design and methods were approved by the Animal Experimentation Committee of the Canton of Vaud, Switzerland (VD3624x1c).

### 2.2. Study Protocol and Lunging Exercise

Owner-reported previous history of respiratory signs and medication in the previous two weeks, current performance and training status were recorded. A complete physical examination including general condition, body weight, body condition score (BCS), heart auscultation, body temperature and mandibular lymph node palpation was performed by one of the authors (JR). The examination of the respiratory system was based on a 23-point weighted clinical score (WCS23), which ranges from 0 (=no clinical signs noted) to a maximum of 23 points, with increasing severity of respiratory clinical signs [15]. Prior to the exercise test, horses were equipped with the monitoring devices and were then led to the lunging area (round pen) where the exercise test took place. Immediately before lunging commenced, pre-exercise respiratory and heart rates (RR and HR, respectively) were recorded. Horses were lunged in a circle with a radius of 6–8 m including three gait phases: 5 min walk, 5 min trot and 5 min canter, changing direction after half of each phase (i.e., after 2.5 min for each gait). HR was monitored in real time via a smartphone application (Polar Sensor Logger for Android, Version 0.31) to ensure horses reached a minimum HR of 100 bpm during the canter phase. Data recording was continuous from one minute before, until 30 min post-exercise. The monitoring device was then removed, and endoscopy and BAL were subsequently performed. The chronological order of the study protocol is illustrated in Supplementary Figure S1.

### 2.3. Monitoring Set-Up and Data Collection

**Figure 1:**
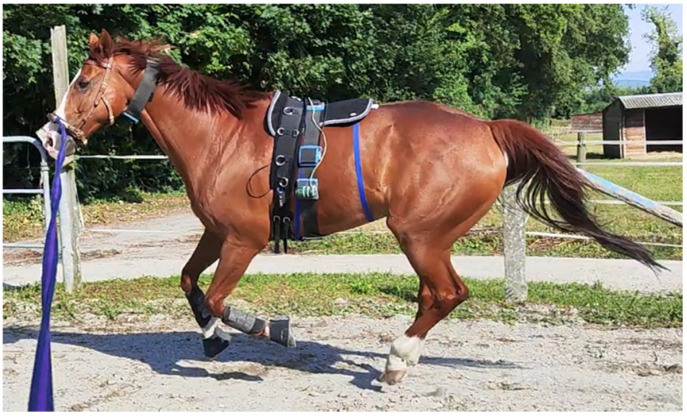
Video still of a horse during lunging exercise wearing the monitoring set-up.

RR, HR and velocity were obtained from a custom-made device that included a global positioning system (GPS) module (u-blox ZED-F9P with a u-blox GNSS multiband antenna ANN-MB-00), a Polar equine belt with the polar device (Polar H10 Heart Rate Monitor, Polar Electro Inc., Kempele, Finland), two plethysmography belts (Open Pleth System, Ambulatory Monitoring Inc., Ardsley, NY, USA) and three wearable digital stethoscopes as described previously [16]. One of the stethoscope heads was placed over the trachea, the other two were placed on either side of the thorax, centrally positioned over the lung field. The polar equine belt was positioned under the lunging girth according to the manufacturer’s recommendations by wetting the horse’s coat and applying contact gel on the left side of the thorax where the belt’s electrodes are located. The front plethysmography girth was placed right behind the lunging girth and the back plethysmography girth at the 15th intercostal space. The device was powered by a battery attached to one of the elastic girths. All modules of the monitoring device were connected to a control unit with USB-C cables, and the device was attached to the horse with elastic belts, as illustrated in Figure 1. Synchronized data were saved on a micro-SD card in the control unit (one csv file for GPS data and one multi-channel 16 bit 4410 Hz PCM WAV file for all other sensors). Ambient temperature and relative humidity were recorded at the lunging area during each test using a digital thermometer and hygrometer (NC-7004-675, Infactory, Pearl GmbH, Buggingen, Germany).

### 2.4. Evaluation of RR, HR, Velocity and Lactate Levels

RR was determined manually from plethysmography data and stethoscope recordings using the software Audacity (Audacity version 3.3.3 for Windows) before exercise, during each gait phase, immediately after ending the exercise and at minutes 1, 2, 5, 10, 15, 20, 25 and 30 post-exercise. One minute of data was analyzed for each timepoint. HR was determined manually from ECG recordings using Kubios HRV (Kubios HRV, University of Eastern Finland, Kuopio, Finland) for the same timepoints as the RR. Velocity was determined from GPS data and evaluated as the median velocity (unit: m/s) for every gait phase, including the changes in direction. Blood samples were collected from the left jugular vein by venipuncture five minutes after finishing the lunging exercise [17,18] and immediately analyzed on-site using a validated portable lactate meter (Lactate Pro 2, Arkray Inc., Siga, Japan) [19].

### 2.5. Endoscopy and BALF-Cytology

Endoscopic examination and BALF collection were performed on the same day, following the exercise test as illustrated in the Supplementary Figure S1. For endoscopic examination and BALF collection, horses were sedated with xylazine (0.3–0.5 mg/kg, IV) and butorphanol (0.01–0.02 mg/kg, IV). The respiratory tract was examined using an endoscope (VET-OR1200HD, Medical Solution GMBH, Wil, Switzerland or SV 60118PKS, Karl Storz GmbH, Tuttlingen, Germany), which was passed through the nasal passage into the trachea down to the carina bifurcation. The amount of mucus present in the trachea was graded using a published scoring system [20]. BALF collection was performed by passing a sterile BALF tube (either 240 or 300 cm in length, depending on the size of the horse, Bivona Medical Technologies, Gary, IN, USA) through the nostril and trachea until it was wedged in a bronchus. While passing the tube, 20 mL of lidocaine (lidocaine 2%, Streuli, Streuli Pharma AG, Uznach, Switzerland) was administered. When the tube was wedged, the cuff was inflated and a volume of 250 mL (in five boluses of 50 mL) sterile 0.9% NaCl was applied using 60 mL syringes and then gently reaspirated. The cuff was deflated, and the BALF tube was removed. The BALF was pooled in a glass bottle and kept refrigerated in EDTA-coated tubes until arriving in the laboratory. All BALF samples were processed the same day as cytospin preparations stained with Wright–Giemsa, and differential cell counts of at least 200 cells were performed at the Veterinary Medical Laboratory of the University of Bern.

### 2.6. Data Categorization and Processing

Breeds were categorized into four groups: 0 = Warmbloods; 1 = Franche-Montagnes Horses; 2 = Thoroughbreds and Standardbreds; 3 = Others (Irish Cob, Friesian, ponies and pony-mixes, Quarter Horses). Training levels were categorized into two categories: 0 = lightly-to-moderately trained for horses exercised 2–4 times per week (including horse-walker, hand walking or riding); 1 = well-trained or highly trained for horses used for dressage, show-jumping, or eventing, exercised > 4 times per week. According to their behavior before, during and after the lunging exercise, horses were classified into two levels of excitement. Excitement level 0 was defined as relaxed behavior before and during exercise and recovery time with only occasional vocalization and standing still during recovery time. Excitement level 1 was defined as marked excitement evidenced by one or several of the following behaviors: raised head, bucking, repeated vocalization or not standing still/pawing the ground during the recovery phase. To ensure consistency, all horses were scored by the same person (JR) immediately after the exercise. For statistical analyses, horses were grouped based on the BALF cytology findings: asthmatic group (>10% neutrophil and/or >5% mast cells and/or >5% eosinophils) and control group (≤10% neutrophils, ≤5% mast cells and ≤5% eosinophils) [1,5,6].

### 2.7. Statistical Analysis

Data were analyzed with the statistic software NCSS (NCSS 2024, LLC. Kaysville, UT, USA, ncss.com/software/ncss) and R (R Foundation for Statistical Computing, Vienna, Austria, R-project.org). The Shapiro–Wilk test was used to assess whether the continuous data followed a normal distribution. Since part of the data was not normally distributed or was categorical, descriptive statistics were summarized using medians and interquartile ranges (IQRs) for continuous data and counts and percentages for categorical data. Pearson’s chi-square or Fisher’s exact test and *t*-tests (Wilcoxon Rank Sum test, Aspen Welch test or Mann–Whitney U test), as appropriate, were used to compare population characteristics between the asthma and control study groups. WCS23, BALF cytology, tracheal mucus scores, environmental temperature and humidity, HR and RR before and during exercise, and velocity during each gait phase were also statistically compared between the study groups. To study the HR and RR response kinetics after the exercise test, we used the difference between individual values at each time point subtracted from the pre-exercise values recorded at the excise site (see Supplementary Figure S1). The resulting delta values (ΔHR and ΔRR) were transformed by square root transformation (ΔHR) and shifted log transformation (ΔRR), respectively, to achieve normality of distribution. The effect of recovery time and study group on ΔHR and ΔRR was analyzed using two-way repeated measures ANOVA.

The odds of the HR and RR not returning to pre-exercise levels within 15 min for variables measured were tested using logistic regression analysis. Associations of HR and RR recovery with asthma status and potential covariates were explored with univariate logistic regression. Potential covariates were sex, age, breed category, BCS, training level, excitement level, WCS23, BALF cellular percentages, tracheal mucus score, average velocity in each gait phase, blood lactate concentration, ambient temperature and humidity. For further multivariate analysis, two of these factors had to be excluded. BALF neutrophil count contributes to the definition of asthma status; therefore, it could not be combined with asthma status in the same regression model. Excitement level was excluded from the analysis for the outcome RR recovery because none of the horses classified as markedly excited had a RR recovery rate <15 min, resulting in extremely large confidence intervals. All potential predictors for the multivariate model were tested for correlation among each other. None of the variables were highly correlated (correlation coefficient r or phi < 0.7). Variables significantly associated with HR and RR recovery, respectively, in the univariate analysis were entered into a multivariate regression model. No variable selection was performed, because only a few variables were significant in univariate analysis. A significance level (α) of 0.05 was used for all statistical tests.

## 3. Results

### 3.1. Study Population Characteristics

Seven horses were excluded because of a history and/or clinical signs of recurrent laryngeal neuropathy (4), inability to complete the full exercise test (2) or poor fit of the monitoring device due to small body size (1). Thirty-seven horses completed the study (21 asthmatic and 16 control horses) (Supplementary Figure S2). Overall (whole population), owner-reported complaints included cough in 26 horses, nasal discharge in 18 horses, increased breathing effort at rest in eight horses, increased breathing effort at exercise in 11 horses and slow recovery after exercise in nine horses. Owners reported discontinuation of medication in two horses in the asthmatic group that received bronchodilator and secretolytics (clenbuterolhydrochloride and dembrexinhydrochloride, orally) for one week up to 48 and 24 h before the exercise test, respectively. Another horse in the asthmatic group received corticosteroids (dexamethasone, IV) and antibiotics (oxytetracycline, IV) for two days up to 72 h before the exercise test. These treatments had been prescribed by the private veterinarian and no further information was available. There were no significant differences in age, sex, BCS, weight, breed categories, training level or excitement level between the two groups (Table 1).

### 3.2. Clinical Findings, WCS23, Tracheal Mucus Score, BALF Cytology, Blood Lactate, Environmental Temperature and Humidity

All horses had a normal body temperature, appetite and were in good general condition. The median HR assessed clinically at rest in the stable for the whole population was 36/min (IQR 4), 36/min (IQR 4) for the control group and 36/min (IQR 5) for the asthmatic group. The median RR at rest in the stable for the whole population was 16/min (IQR 8), 16/min (IQR 8) for the control group and 16/min (IQR 10) for the asthmatic group. Resting HR and RR at the stable did not differ significantly between the groups.

WCS23 scores ranged from 0 to 11 overall (whole population median 2, IQR 2). The highest score in the control group was 3 (median 2, IQR 1), the highest score in the asthmatic group was 11 (median 3, IQR 2; *p* = 0.004). As shown in Table 2, the asthmatic group exhibited higher neutrophil percentages in BALF (*p* < 0.001), while eosinophil and mast cell percentages did not differ between groups. Tracheal mucus scores were higher in the asthmatic group (*p* = 0.03), but lactate values did not differ between groups. Tracheal mucus scores (*p* = 0.03) and BALF neutrophil percentages (*p* < 0.001) were increased in the asthmatic compared to the control group (Table 2). Cytologic findings of the BALF can be found in Table 2.

The median lactate values 5 min after exercise for the whole population were 1.5 mmol/L (IQR 1.4), and did not differ between the control group (median 1.4 mmol/L, IQR 1.3) and the asthmatic group (median 1.7 mmol/L, IQR 2.2). The median environmental temperature across all exercise tests (whole population) was 15.0 °C (IQR 11.3), 19.5 °C (IQR 12.6) for the control group and 15.0 °C (IQR 10.0) for the asthmatic group. The median environmental humidities for the whole population were 65% (IQR 10.0), 65% (IQR 7.8) for the control group and 64% (IQR 12.0) for the asthmatic group. These environmental factors did not differ between groups.

### 3.3. HR, RR and Velocity Before Exercise and During Each Gait Phase

The results for HR, RR and velocity before and during exercise for the entire study population, the control and the asthmatic group, are presented in Table 3. HR data quality during exercise was insufficient for analysis in three horses, and pre-exercise RR data quality was insufficient in two other horses as well as HR in one horse. No significant differences were found between the groups for all these variables.

### 3.4. HR and RR Recovery After Exercise over Time

**Figure 2:**
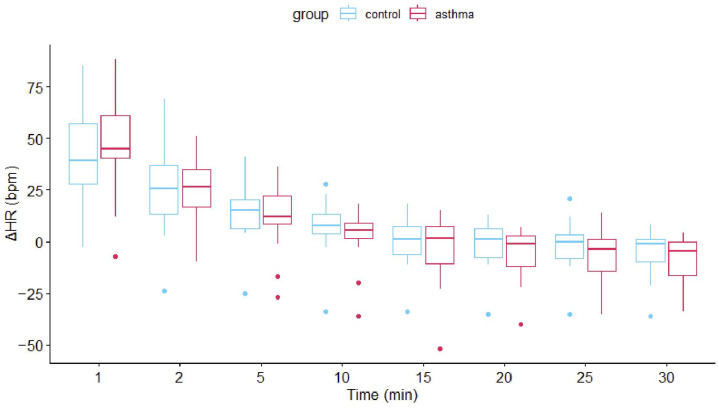

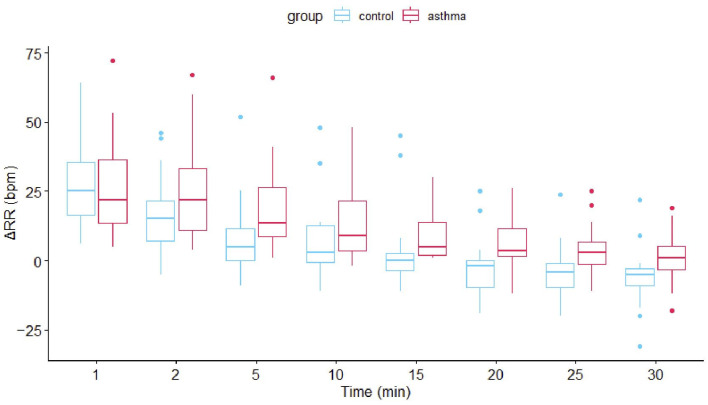
Box- and whisker plots with outliers comparing changes in post-exercise ΔHR (bpm = beats per minute; upper figure) and ΔRR (bpm = breath per minute; lower figure) between groups. Δ indicates time point values subtracted from pre-exercise baseline values at each time point (in minutes).

There was a significant effect of time on ΔHR and ΔRR (*p* < 0.001) in the repeated measures ANOVA. Both variables decreased over time. Asthma also significantly affected ΔRR with lower values in the asthmatic group (*p* < 0.001). However, while both time and group had significant effects on ΔRR, the changes over time were similar between the two groups (non-significant interaction term). Recovery after exercise is shown in Figure 2.

### 3.5. Regression for HR and RR Recovery to Baseline

Univariate binomial logistic regression of the recovery time (≤15 vs. >15 min) required to return to pre-exercise HR and RR showed several significant findings (Supplementary Table S3). The age and the level of training significantly predicted HR, but not RR recovery time. HR recovery was longer in older horses (OR 1.20, 95% CI 1.03–1.40, *p* = 0.02), and shorter in horses with a higher training level (OR 0.14, 95% CI 0.02–0.83, *p* = 0.03). Combining the two predictors in a multivariate model resulted in smaller and non-significant effects, likely because older horses had a lower training level (correlation coefficient—0.37; *p* = 0.02). RR recovery time was significantly longer in asthmatics than in controls (OR 52.25, 95% CI 5.17–528.28, *p* < 0.001) and in horses with a higher BALF neutrophil count (OR 1.14 per percent increase, 95% CI 1.01–1.29, *p* = 0.04), while mast cell and eosinophil percentages as well as the amount of tracheal mucus had no significant effect. The trot velocity was also found to significantly increase the time taken to return to the initial RR (OR 17.32, 95% CI 1.23–244.15, *p* = 0.03). In the multivariate model with asthma status and trot velocity (correlation coefficient 0.24; *p* = 0.16) as predictors of RR recovery, only the asthma status remained significant. The other covariates, sex, breed, BCS, WCS23, blood lactate concentration, velocity at the other gaits during exercise, as well as environmental factors (ambient temperature and humidity), had no significant influence on HR or RR recovery.

For visualization, the effect of training level on HR recovery time is illustrated as the proportions of horses in each group of training level and categorized into ≤15 and >15 min HR recovery time (Supplementary Figure S4). Correspondingly, Supplementary Figure S5a illustrates the proportions of horses in the control vs. asthmatic group categorized into ≤15 and >15 min RR recovery time. Supplementary Figure S5b illustrates the same proportions, when the three horses that received medication within two weeks before the exercise test are excluded from the asthmatic group. The proportions of ≤15 vs. >15 min RR recovery time in the asthmatic group were almost identical whether the three horses were included or not.

## 4. Discussion

This study shows that equine asthma can affect recovery from submaximal exercise in leisure and sport horses. Horses with neutrophilic airway inflammation classified as asthmatics were more likely to take longer than 15 min for their RR to return to pre-exercise values. This aligns with the original description of the submaximal exercise test [13], on which our protocol and hypothesis were based. However, in contrast to RR, HR recovery was affected by the training level and age, but not by cytological signs of equine asthma, suggesting cardiovascular fitness plays a larger role in HR response. These findings support the utility of submaximal lunging tests for detecting respiratory limitations in asthmatic leisure and sport horses.

The monitoring equipment was well tolerated by all horses and provided valuable information on variables of interest such as HR, RR and velocity during exercise. HR was based on the Polar device, offering real-time feedback during lunging. Analyses from the recordings were undertaken to enhance the accuracy of measurements, in contrast to the originally described clinical test, which depends on the clinician’s post-exercise assessment based on auscultation of HR and observation of RR [13]. The monitoring equipment providing recordings of good quality enabled us to demonstrate that HR, RR and velocities across all gait phases showed no significant differences between groups, suggesting that the effort was comparable for both asthmatic and control horses. However, the original test’s simplicity and clinical applicability justify further exploration to assess whether clinical monitoring of recovery following submaximal lunging exercise could serve as a straightforward indicator of functional deficits linked to equine asthma.

Unlike our findings, Beling et al. [11] observed no delayed HR or RR recovery in horses with cytological signs of asthma during post-exercise testing. However, all these horses were asymptomatic, and their protocol involved stricter cytological cutoffs. Also, RR assessments were limited to only two time points post-exercise (immediately—and 30 min after the test), potentially overlooking delayed recovery which was most evident in our intermediate time points at 15 min post-exercise.

Asthma status, which was of the neutrophilic type in our cohort, was the primary predictor of delayed RR recovery, consistent with previous studies linking neutrophilic inflammation to impaired performance in racehorses [4,5,21] and in endurance horses [9]. Interestingly, our data showed no association of mast cell and eosinophil percentages with RR recovery, diverging from findings where increased mast cells contributed to performance deficits in racehorses [8]. This discrepancy may reflect the low prevalence of mastocytic or eosinophilic inflammation in our population. While mastocytic asthma has been linked to airway hyperreactivity in horses presenting with poor performance [22], neutrophilic inflammation appears to be the dominant driver of impaired recovery in submaximal exercise, at least within the cohort examined. Larger studies may be necessary to determine the significance of non-neutrophilic inflammation on RR recovery.

Despite higher tracheal mucus scores in asthmatic horses, we found no effect of mucus accumulation on HR or RR recovery. Studies on tracheal mucus and poor performance show mixed results. While some report no effects of tracheal mucus accumulation on race performance in thoroughbreds [5,23], others observed associations between decreased performance and increased tracheal mucus accumulation in thoroughbred racehorses [24], standardbred trotters [25] and endurance horses [9]. Also, dressage and show-jumping horses with increased tracheal mucus were less willing to perform [26]. Still, the present findings suggest that airway inflammation, rather than mucus accumulation, plays a more critical role in delayed RR recovery. Still, the present findings suggest that airway inflammation, rather than mucus accumulation, plays a more critical role in delayed RR recovery.

The underlying pathophysiological mechanisms leading to decreased athletic performance in equine asthma are not fully understood. Bronchoconstriction, airway hyperresponsiveness, airway remodeling and impaired pulmonary gas exchange are documented in equine asthma [1] and could influence pulmonary function during and after exercise. Herteman et al. [14] observed airflow changes in horses 15 min post exercise measured by electrical impedance tomography using the same lunging protocol as the present study. Lo Feudo et al. [6] also reported lung function differences between healthy controls and horses with mild-to-moderate equine asthma 15 min post-exercise using respiratory oscillometry following a more intensive exercise test. Both studies discuss bronchoconstriction as an important potential cause for the observed respiratory limitations in asthmatic horses after exercise. Bronchoconstriction has also been suggested as a potential explanation for the prolonged RR recovery times after exercise in children with uncontrolled asthma compared to those with controlled asthma [27]. In the present study, the short time defined to discontinue medication could have influenced the results, but all three horses in question belonged to the asthmatic group. Accordingly, potential consequences of the medication would be expected to diminish the observed effect of asthma on RR recovery. However, comparison of the proportions of asthmatic horses categorized into ≤15 and >15 min RR recovery time showed only a negligible difference when the three horses were excluded (Supplementary Figure S5a,b). Further research involving controlled pharmacological interventions to induce bronchodilatation in asthmatic horses during and after the exercise could provide valuable insights.

Lactate values during and after exercise reflect the anaerobic effort and are used to assess aerobic capacity in horses [28,29]. In the present study, post-exercise lactate concentrations were low in both asthmatic and control horses, indicating minimal anaerobic effort during the lunging test. This aligns with a previous study reporting low lactate levels in horses during submaximal exercise [28]. In contrast, high-intensity protocols revealed elevated lactate levels 5 min after exercise in asthmatic horses [5,9,17]. These discrepancies can be attributed to the relatively low intensity of our exercise protocol. Our population was composed of leisure and sport horses working at substantially lower speeds than racehorses. Consequently, the median velocities of our exercise protocol, even at the canter, were low. Nevertheless, moving in a circle is expected to be more demanding for the horses than working in a straight line [30]. While we specifically chose this submaximal exercise as it is a method described for clinical practice [13], the protocol may not be sufficiently strenuous to detect cardiovascular effects, such as delayed HR recovery, especially in milder cases of equine asthma. HR recovery, in contrast to RR, was not influenced by asthma status, but decreased with higher training level and increased with age, highlighting the importance of cardiovascular conditioning in moderating post-exercise responses, especially for older horses. This mirrors findings in athletic horses, where HR recovery reflects fitness and exercise intensity [30]. The absence of asthma-related effects on HR recovery suggests that cardiovascular adaptations can compensate for mild respiratory limitations.

This study’s small sample size and the number of potential influencing factors present certain limitations. Although asthma status strongly predicted RR recovery, the wide confidence intervals reflect potential uncertainty and overestimated effect sizes due to the limited cohort size. We acknowledge the risk of overfitting and limited statistical power in our regression models. The findings should therefore be interpreted with caution, and further studies with larger sample sizes are needed to validate these results. We adopted the broader cytological cutoffs for asthma classification [1,5,6], which assured balanced group representation. Significant differences in clinical scores between asthmatic and control horses underscore the robustness of our classification approach. However, most clinical signs were relatively mild, and severely affected horses were excluded from the study as they would not have been able to complete the lunging exercise.

Air quality and allergen concentration were not monitored in our study and may have influenced the performance and recovery of the horses. Exposure to irritants like dust and mold can exacerbate airway inflammation, affecting respiratory function [8,31]. The influence of these variables on performance and recovery time would be an interesting question for future studies. Other key intrinsic and extrinsic confounders were well balanced between the two groups and accounted for in the statistical analysis. Ranges of some of the potential confounders were limited, however. For instance, BCS ranged from 4 to 7, reflecting the absence of horses that were either markedly over- or underweight. We anticipated that excitement levels might influence vital parameters during and after exercise, as HR, particularly below 160 bpm, can be affected by individual excitement during submaximal exercise [30,32]. Yet, while training status significantly influenced HR recovery, excitement level did not affect HR in this study.

## 5. Conclusions

Equine asthma could be a predictor for delayed RR recovery. Higher training level improved HR recovery, suggesting that cardiovascular fitness plays a larger role in HR response. These findings highlight the potential of submaximal lunging exercise testing as a practical tool for identifying respiratory limitations in asthmatic leisure and sport horses. Further research in larger populations is necessary to explore RR recovery as a non-invasive marker for equine asthma and its impact on athletic performance.

## Figures and Tables

**Table 1:** Population characteristics.

	Whole Population (*n* = 37) Median [IQR] or Number (%)	Control Group (*n* = 16) Median [IQR] or Number (%)	Asthmatic Group (*n* = 21) Median [IQR] or Number (%)
Age (years)	14.0 [9.5]	12.5 [8.8]	16.0 [9.5]
Weight (kg)	515.0 [105.5]	513.5 [117.8]	515.0 [94.0]
BCS (1–9)	5.0 [1.0]	5.5 [1.0]	5.0 [1.0]
Sex			
Mare	14 (37.8%)	8 (50%)	6 (28.6%)
Gelding	18 (48.7%)	6 (37.5%)	12 (57.1%)
Stallion	5 (13.5%)	2 (12.5%)	3 (14.3%)
Breed			
Warmblood	18 (48.7%)	10 (62.5%)	8 (38.1%)
Franches-Montagne	7 (18.9%)	2 (12.5%)	5 (23.8%)
Standardbred or Thoroughbred	3 (8.1%)	1 (6.3%)	2 (9.5%)
Others	9 (24.3%)	3 (18.7%)	6 (28.6%)
Training level 0	28 (75.7%)	13 (81.2%)	15 (71.4%)
Training level 1	9 (24.3%)	3 (18.8%)	6 (28.6%)
Excitement level 0	29 (78.4%)	14 (87.5%)	15 (71.4%)
Excitement level 1	8 (21.6%)	2 (12.5%)	6 (28.6%)

Numerical values are shown as medians and interquartile ranges (IQRs), categorical values are shown as counts and percentages. No significant differences were found for all variables between the study groups (*t*-test for continuous variables or Fisher test for categorical variables), with all *p*-values above 0.05. Body condition score (BCS). Training level 0 = untrained to moderately trained; training level 1 = well-trained to highly trained. Excitement level 0 = calm behavior to mild excitement; excitement level 1 = marked excitement.

**Table 2:** Endoscopic (tracheal mucus score) and bronchoalveolar lavage (BALF) cytology findings.

	Whole Population (*n* = 37) Median [IQR]	Control Group (*n* = 16) Median [IQR]	Asthmatic Group (*n* = 21) Median [IQR]
BALF cytology			
Neutrophils (%)	10 [17.5]	5.0 [4.8]	17.3 [24.8] *
Mast cells (%)	2.0 [2.7]	2.0 [1.5]	2.0 [5.5]
Eosinophils (%)	0.0 [0.5]	0.0 [0.5]	0.0 [0.5]
Macrophages (%)	38.3 [16.8]	39.2 [20.9]	35.0 [15.5]
Lymphocytes (%)	44 [26.3]	51.3 [15.5]	39.0 [22.0] *
Tracheal mucus score (0–5)	2.0 [2.0]	1.0 [3.0]	3.0 [2.0] *

Data are presented as medians and interquartile ranges (IQRs). Tracheal mucus was scored on a scale from 0 to 5 (Gerber et al. 2004b). * Indicate significant differences from controls (*p* < 0.05) for the other variables no significant differences were found between the study groups (*t*-test for continuous variables or Fisher test for categorical variables), with *p*-values above 0.05.

**Table 3:** Heart rate, respiratory rate and velocity before and during exercise.

	Whole Population (*n* = 37) Median [IQR]	Control Group (*n* = 16) Median [IQR]	Asthmatic Group (*n* = 21) Median [IQR]
Heart rate pre-exercise (/min)	44.5 [19.8] (*n* = 36)	45.0 [15.3] (*n* = 16)	44.5 [27.0] (*n* = 20)
Heart rate walk (/min)	74.0 [25.5] (*n* = 34)	72.0 [17.0] (*n* = 15)	78.0 [28.0] (*n* = 19)
Heart rate trot (/min)	114.0 [36.0] (*n* = 34)	112.0 [32.0] (*n* = 15)	118.0 [35.0] (*n* = 19)
Heart rate canter (/min)	125.0 [34.3] (*n* = 34)	121.0 [19.0] (*n* = 15)	140.0 [48.0] (*n* = 19)
Respiratory rate pre-exercise (/min)	28.0 [17.0] (*n* = 35)	32.0 [17.0] (*n* = 15)	27.5 [20.3] (*n* = 20)
Respiratory rate walk (/min)	53.0 [11.5] (*n* = 37)	52.0 [12.5] (*n* = 16)	54.0 [12.5] (*n* = 21)
Respiratory rate trot (/min)	79.0 [8.0] (*n* = 37)	77.0 [4.0] (*n* = 16)	81.0 [10.0] (*n* = 21)
Respiratory rate canter (/min)	104.0 [9.0] (*n* = 37)	104.0 [9.5] (*n* = 16)	107.0 [7.0] (*n* = 21)
Velocity walk (m/s)	1.2 [0.2] (*n* = 37)	1.2 [0.1] (*n* = 16)	1.2 [0.3] (*n* = 21)
Velocity trot (m/s)	2.4 [0.5] (*n* = 37)	2.3 [0.2] (*n* = 16)	2.5 [0.7] (*n* = 21)
Velocity canter (m/s)	3.3 [0.8] (*n* = 37)	3.1 [0.6] (*n* = 16)	3.5 [0.8] (*n* = 21)

Values are shown as median and interquartile range (IQR), n indicates number of horses included in the respective analyses since measurements of insufficient quality were removed. No significant differences were found for any variables between the study groups (*t*-test for continuous variables), with all *p*-values above 0.05.

## Data Availability

Raw data file is openly available in Zenodo (https://doi.org/10.5281/zenodo.14619627, accessed on 15 January 2025).

## Supplementary Materials

The following supporting information can be downloaded at: https://www.mdpi.com/article/10.3390/ani15050713/s1; Figure S1: Chronological study protocol; Figure S2: Study population, excluded horses and group allocation; Table S3: Summary table logistic regression; Figure S4: Heart rate recovery ≤15 and >15 min (min) for training level groups (proportions, %); Figure S5: a: Respiratory rate recovery categorized into ≤15 and >15 min (min) for asthma groups (proportions, %); b: Respiratory rate recovery categorized into ≤15 and >15 min (min) for asthma groups (proportions, %)—3 horses excluded because of recent medication.
