# Supplementary material for: Respiratory Rate Recovery After Submaximal Lunging Exercise Is Delayed in Asthmatic Horses with Neutrophilic Airway Inflammation
Source: Animals (Basel). 2025 Mar 2;15(5):713. doi: 10.3390/ani15050713 (PMC11899412; doi:10.3390/ani15050713)
Supplement: Supplementary file 1 [file animals-15-00713-s001.zip › Supplementary Figure S1.pdf]

Supplementary Figure S1. Chronological study protocol.

Abbreviations: HR = heart rate; RR = respiratory rate; BCS = body condition score;
WCS23 = weighted clinical score; BALF = bronchoalveolar-lavage fluid

Clinical examination at the stable

Complete physical examination (clinically assessed HR and RR, general condition, body weight, BCS, heart auscultation, body temperature, mandibular lymph node palpation) and completion of WCS23

↓ Attaching the measurement devices and leading the horse to exercise location

Exercise test

Monitoring of HR, RR and velocity immediately before exercise, during 15 minutes of lunging exercise and up to 30 minutes after exercise – recording of ambient temperature and humidity at location

Lactate sample collection 5 minutes after ending the lunging exercise

↓ Leading the horse back to the stable and removing the equipment; leading the horse to the examination room

Endoscopic examination including mucus scoring and BALF-collection
