# Supplementary material for: Respiratory Rate Recovery After Submaximal Lunging Exercise Is Delayed in Asthmatic Horses with Neutrophilic Airway Inflammation
Source: Animals (Basel). 2025 Mar 2;15(5):713. doi: 10.3390/ani15050713 (PMC11899412; doi:10.3390/ani15050713)

Supplementary Figure S4. Heart rate recovery ≤ 15 and >15 minutes (min) for training level groups (proportions [%] of 9 horses with training level 1 and 27 horses with training level 0, respectively).

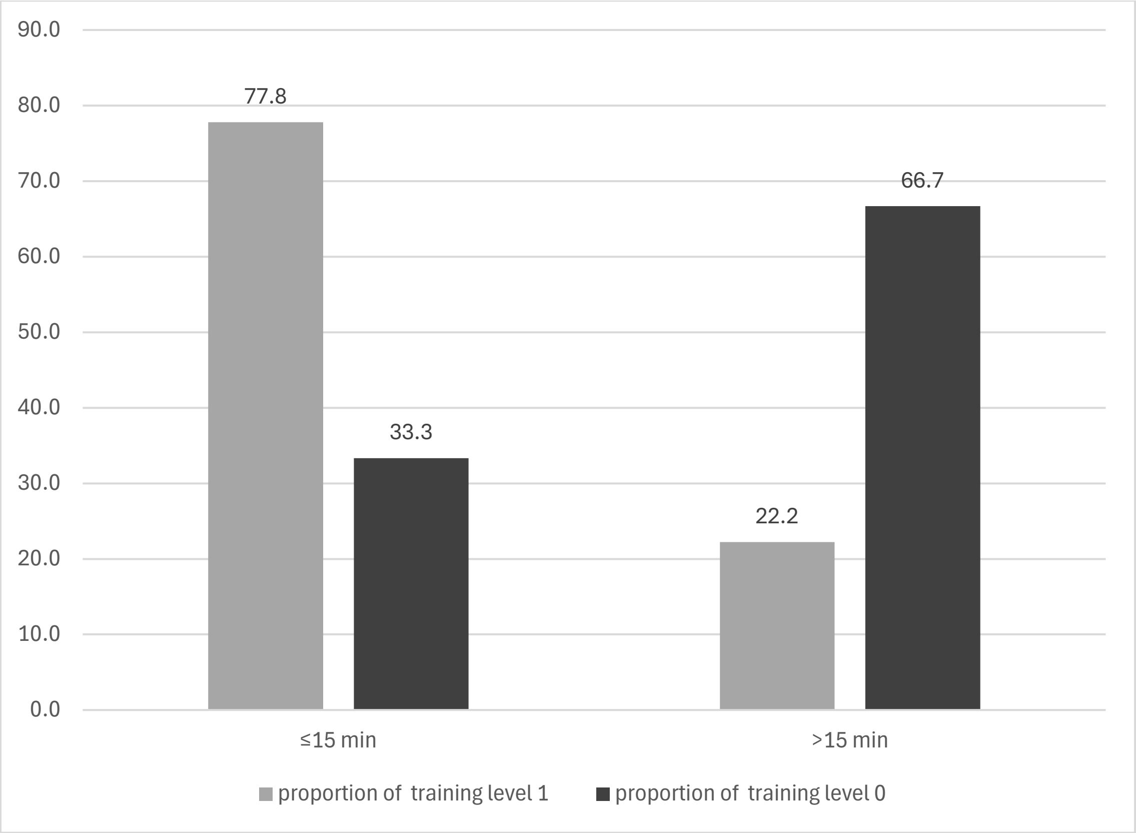

Supplement: Supplementary file 1 [file animals-15-00713-s001.zip › Supplementary Figure S4.pdf]
