# Supplementary material for: Respiratory Rate Recovery After Submaximal Lunging Exercise Is Delayed in Asthmatic Horses with Neutrophilic Airway Inflammation
Source: Animals (Basel). 2025 Mar 2;15(5):713. doi: 10.3390/ani15050713 (PMC11899412; doi:10.3390/ani15050713)

Supplementary Figure S5b. Respiratory rate recovery categorized into ≤ 15 and >15 minutes (min) for asthma groups (proportions, [%] of 15 controls and 17 asthmatics, respectively) - 3 horses were excluded because of recent medication.

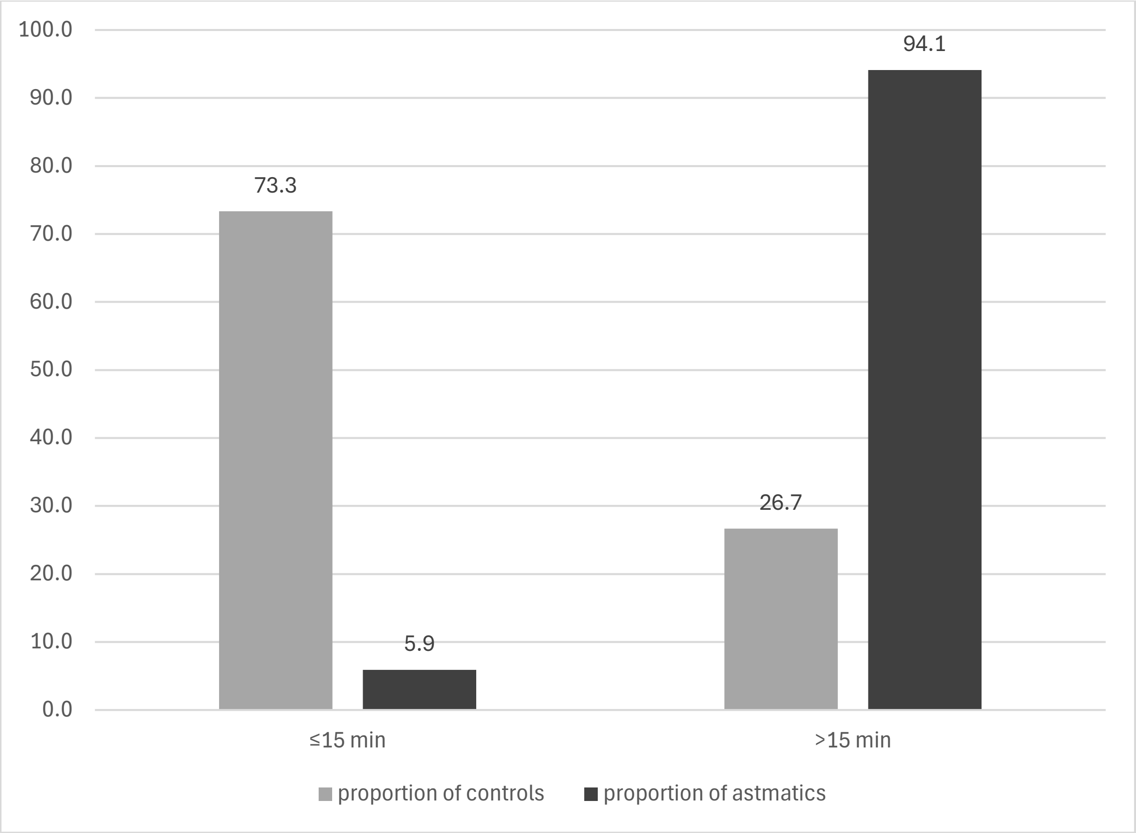

Supplement: Supplementary file 1 [file animals-15-00713-s001.zip › Supplementary Figure S5b.pdf]
