# Supplementary material for: Respiratory Rate Recovery After Submaximal Lunging Exercise Is Delayed in Asthmatic Horses with Neutrophilic Airway Inflammation
Source: Animals (Basel). 2025 Mar 2;15(5):713. doi: 10.3390/ani15050713 (PMC11899412; doi:10.3390/ani15050713)
Supplement: Supplementary file 1 [file animals-15-00713-s001.zip › Supplementary Table S3.pdf]

Supplementary Table S3. Summary table logistic regression.

Model	Outcome	Predictor	Estimate	OR	95%	Cl	p-value
**Univariate**	**HR recovery time**	Age	0.19	1.20	1.03	1.40	**0.02**
		Sex	0.61	1.83	0.43	7.77	0.41
		Breed_category	-0.17	0.84	0.14	4.97	0.85
		BCS	-1.01	0.36	0.03	4.74	0.44
		Training_level	-1.95	0.14	0.02	0.83	**0.03**
		Asthma	-0.05	0.95	0.25	3.57	0.94
		WCS23	-0.81	0.44	0.03	6.70	0.56
		Tracheal mucus	-1.95	0.14	0.02	0.83	0.48
		BAL_neutrophils	0.02	1.02	0.97	1.06	0.44
		BAL_eosinophils	0.50	1.65	0.62	4.40	0.31
		BAL_mastcells	-0.06	0.94	0.70	1.27	0.69
		Temperature	0.09	1.10	0.98	1.23	0.11
		Humidity	0.10	1.10	0.98	1.24	0.11
		Excitement_level	-0.950	0.39	0.08	1.96	0.25
		Lactate_venous	0.00	1.00	0.67	1.50	1.00
		Velocity_walk_median	1.03	2.80	0.08	95.59	0.57
		Velocity_trot_median	0.49	1.64	0.33	8.02	0.54
		Velocity_canter_median	0.01	1.01	0.41	2.49	0.99
**Multivariate**		Age	0.154	1.17	0.99	1.37	0.063
		Training_level1	-1.4762	0.23	0.04	1.47	0.121
**Univariate**	**RR recovery time**	Age	0.11	1.12	0.97	1.29	0.12
		Sex	0.02	1.02	0.23	4.47	0.98
		Breed_category	0.66	1.94	0.29	13.19	0.50
		BCS	-0.09	0.92	0.07	12.32	0.95
		Training_level	-0.18	0.83	0.16	4.30	0.83
		Asthma	3.96	52.25	5.17	528.28	**0.0008**
		WCS23	-1.79	0.17	0.01	2.82	0.21
		Tracheal mucus	-0.88	0.42	0.05	3.31	0.41
		BAL_neutrophils	0.13	1.14	1.01	1.29	0.04
		BAL_eosinophils	0.26	1.29	0.54	3.08	0.56
		BAL_mastcells	0.30	1.35	0.88	2.07	0.17
		Temperature	-0.07	0.94	0.83	1.06	0.29
		Humidity	0.02	1.02	0.92	1.15	0.67
		Lactate_venous	0.77	2.16	0.89	5.25	0.09
		Velocity_walk_median	4.64	103.45	0.06	17937.18	0.08
		Velocity_trot_median	2.85	17.32	1.23	244.15	**0.03**
		Velocity_canter_median	0.76	2.14	0.64	7.17	0.22
**Multivariate**		Asthma	4.12	61.56	4.20	902.53	**0.003**
		Velocity_trot_median	3.58	35.87	0.54	2378.92	0.09

Results of binomial logistic regression with the outcome time to return to baseline for HR and RR (recovery time ≤15min vs. >15min) and predictors of interest. Significant p-values are marked in bold.
Category references: Sex (Sex 1 = mare, Sex 2 = gelding, Sex 3 = stallion); Breed categories (Breed cat. 1 = Warmbloods, Breed cat. 2 = Franche-Montagnes Horses, Breed cat. 3 = Standardbreds or Thoroughbreds, Breed cat. 4 = Others); BCS (Body Condition Score 1-9); untrained to highly trained (training level 0 = not trained or very lightly trained, training level 1 = well-trained to highly trained); asthma vs. control; WCS23 (weighted clinical score 0-23); Tracheal mucus (0-5); relaxed behaviour vs. marked excitement (excitement level 0 = relaxed behaviour, excitement level 1 = marked excitement)
