# Supplementary figures and images for: Respiratory Rate Recovery After Submaximal Lunging Exercise Is Delayed in Asthmatic Horses with Neutrophilic Airway Inflammation
Source: Animals (Basel). 2025 Mar 2;15(5):713. doi: 10.3390/ani15050713 (PMC11899412; doi:10.3390/ani15050713)

**Supplementary Figure S2.** Study population, excluded horses and group allocation.

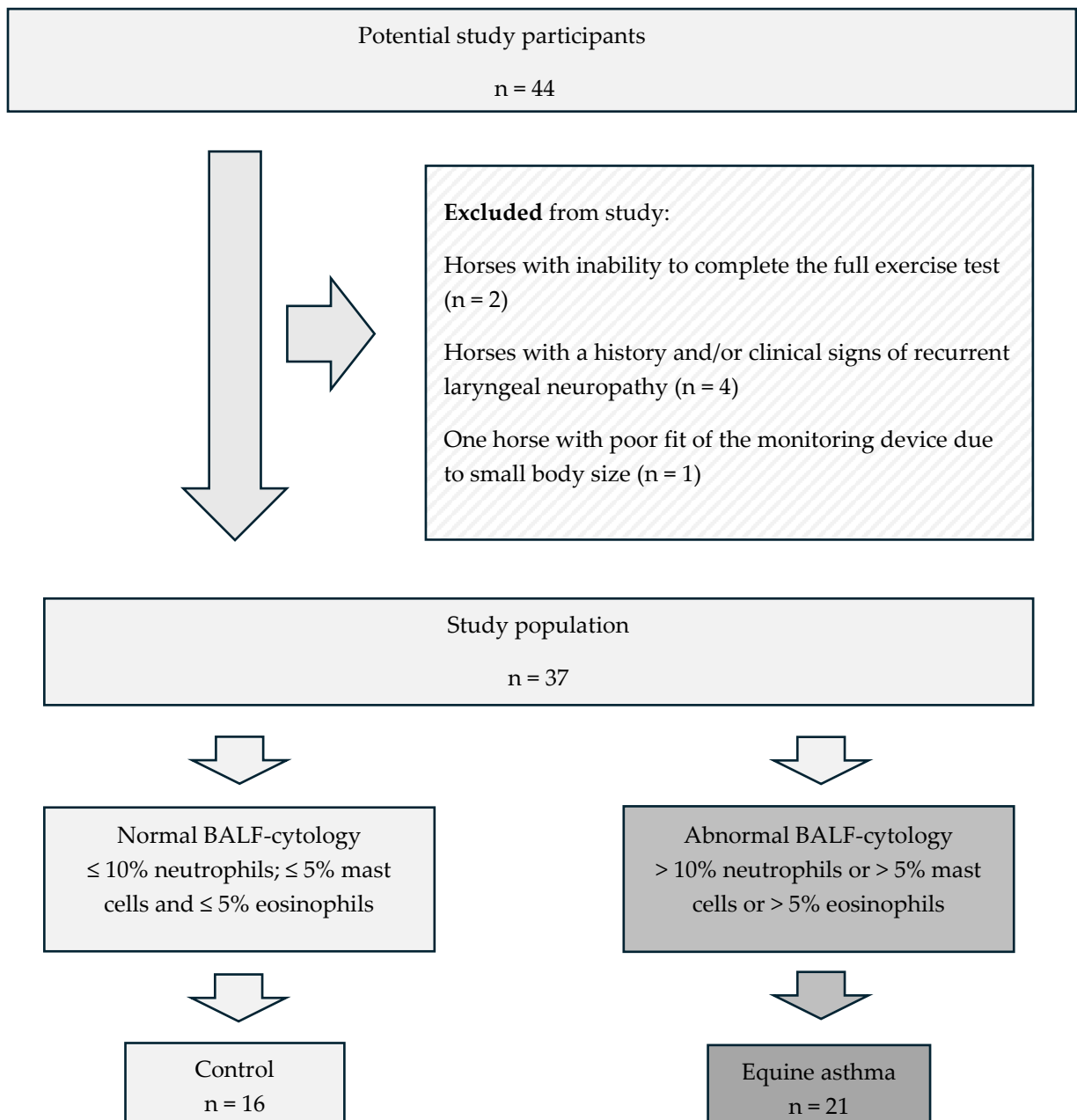

Supplement: Supplementary file 1 [file animals-15-00713-s001.zip › Supplementary Figure S2.pdf]
